# Isolation and screening of actinomycetes producing antimicrobial substances from an extreme Moroccan biotope

**DOI:** 10.11604/pamj.2019.33.329.19018

**Published:** 2019-08-29

**Authors:** Abdenbi El Karkouri, Soumia Ait Assou, Mohammed El Hassouni

**Affiliations:** 1 Team of Biotechnology and Environment, Natural Resources and Environment Laboratory, Department of Biology Chemistry Geology, Polydisciplinary Faculty of Taza, Sidi Mohamed Ben Abdellah University, Fez 30050, Morocco; 2 Team of Microbial Biotechnology, Biotechnology Laboratory, Faculty of Sciences Dhar El Mahraz, Department of Biology, Sidi Mohamed Ben Abdellah University, Fez 30050, Morocco

**Keywords:** Actinomycetes, antimicrobial assay, saline environment, Actinomycetes, antimicrobial assay, saline environment

## Abstract

**Introduction:**

This study was carried out to isolate and screen actinomycetes from soil of two salterns in Taza-Morocco, for the production of antimicrobial compounds against a set of target bacteria. Also, it aims to highlight some practices in order to isolates actinomycetes and screen for their ability to produce antibacterial compounds.

**Methods:**

Soil samples were analyzed for physical and chemical parameters including pH, electrical conductivity, and salinity. The actinomycetes were isolated on Casein Starch Agar (CSA) medium and purified on International Streptomyces Project 2 (ISP-2) medium. Antimicrobial activity of actinomycete isolates was evaluated by measuring the inhibition zone. These activities were tested against Dickeya solani IP2222, Pectobacterium brasiliensis 13471a, Escherichia coli K12, Proteus mirabilis, Pseudomonas aeruginosa CECT118, Listeria innocua CECT4030, Staphylococcus aureus CECT976, Bacillus subtilis DSM 347 and Candida alibicans, using three different culture media (CSA, Bennett and Mueller Hinton) and at two temperatures of incubation (30°C and 37°C).

**Results:**

Physical and chemical analysis of soil samples showed that both sites are alkaline. Also, with regards to salinity, the second site showed to contain high salt concentration compared the first site. The abundance of bacteria isolated on CSA medium from both sites showed correlation with the physical-chemical properties of the sampling soils. Incubation temperature of 30°C resulted in a high number of actinomycetes (18/22) isolates with antimicrobial effect relative to the temperature of 37°C (4/22). Some actinomycetes isolates show antimicrobial effect on only one culture medium, which shows a special nutritional requirement to express their antimicrobial effect. On the other hand, some isolates, they express their antimicrobial effect on the three media at the same time. Additionally, some isolates of actinomycetes inhibit the growth of several microorganisms at once. While others inhibit the growth of only one microorganism tested which reflects a possible specificity of antimicrobial substances.

**Conclusion:**

Growth conditions including, media composition, temperature of incubation and the spectrum of test strain tailors the behavior of the antimicrobial screening.

## Introduction

Actinomycetes are Gram-positive filamentous bacteria with fungal morphology. They are widely distributed in nature, particularly in soil [1]. They constitute a significant portion of the telluric microflora (10^+4^10^+6^ CFU/ml) and (10^+7^W22;10^+8^ CFU/ml) [2]. Actinomycetes are widespread in nature and may occur in extreme environments [3]. Microorganisms found in extreme environments have attracted a great deal of attention, due to the production by such microorganisms of various natural compounds and their specialized mechanisms for adaption to extreme environments [4]. Among the various extremophiles, halophilic microorganisms have developed several strategies to survive and to function in hypersaline ecosystems, such as salterns, salt mines and other hypersaline environments [5]. The cultivation of actinomycetes from extreme environments including saline habitats is very difficult than common environments, because of their slow growth rate. In the conventional isolation techniques, several factors must be considered, namely, the choice of screening source, the selective medium, culture conditions and the recognition of candidate colonies in the primary isolation. Furthermore, choosing appropriate media and growth conditions is important and published media are typically associated with a particular microbial genus or species. As with other microbial discovery research, when working with environmental samples harboring communities of novel microbial populations, the media and growth conditions chosen will enrich for certain populations and not others [6]. Actinomycetes are known as the most biotechnologically valuable prokaryotic microorganisms. They are well known as a source of antibiotics and bioactive molecules. Most of their bioactive molecules have been shown to have antibacterial (streptomycin, tetracycline and chloramphenicol), antifungal (nystatin), antiviral (tunicamycin) and antiparasitic (avermectin) properties [7]. Indeed, most of the antimicrobials used today in remedying diseases caused by pathogens have been developed from actinomycetes. Currently, over 5000 antibiotics have been screened from Gram positive, Gram negative bacteria as well as fungi. However, only 100 of these antibiotics have been developed to clinical applications [8]. Nowadays, the control of pathogenic microorganisms by synthetic products is becoming less attracting due to the emergence of resistant strains and because of the undesirable effects of these products on the environment. Therefore, it's absolutely necessary to find antagonistic microorganisms that are used as a means of bio-control. In this study we were interested in isolating actinomycetes from two Moroccan saline soils and screening of isolates for the production of antimicrobial substances in three culture media of different compositions (Casein Starch Agar (CSA), Bennett's agar, Mueller-Hinton (MH) agar) and at two incubation temperatures (30°C and 37°C).

## Methods

**Figure 1:**
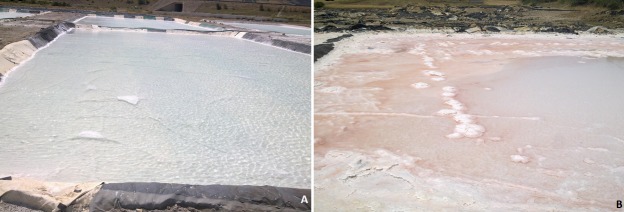
Basins evaporation of saline water, (A) site number 1 and (B) site number 2

**Studied sites:** the sampling sites in this study were soil of a saltern (Figure 1). Soil samples were collected from two locations in the valley of Taza, Morocco. The GPS coordinates of the two sites (site 1 (Figure 1 A) and site 2 (Figure 1 B) are 4°03'W and 34°15'N at altitude of 418m and 4°06'W and 34°13'N at altitude of 385m. The weather is typically Mediterranean, semi-arid to arid, with an average rainfall of 517mm per year and an average annual temperature of 18 - 21°C.

**Sampling:** from each site, 5 soil samples were collected aseptically in sterile plastic bags from the 10cm upper layer of soil at five random locations and transported to the laboratory.

**Soil chemical analysis:** we were interested in determining the electrical conductivity (EC) and pH of soil samples. CONSORT-Model C535 (μs/cm, pH)-G. BOYER-S/N 71658 was used in this regard. For samples preparation for pH determination, 10g of soil sample were mixed with 50ml of distilled water. After agitating for 15 minutes, the suspension was allowed to settle for 2 h [9]. For the measurement of the EC, 5g of the soil were mixed with 25ml of distilled water. After agitating for 30 min, the suspension was allowed to settle for 15 min and centrifuged (3000 rpm/10min) [10]. Each analysis was carried out in triplicate. The electrical conductivity is expressed according to the formula: EC 25°C (dS/m ) = ECt×Ft. ECt: conductivity at temperature of extract & Ft: temperature coefficient.

**Media:** the Casein Starch Agar (CSA) medium used for actinomycetes isolation contained (soluble starch 10g, casein 0.3 g, KNO 2 g, NaCl 2 g, K_2_HPO_4_ 2 g, MgSO_4_-7H_2_O 0.05 g, CaCO_3_ 0.02 g, FeSO_4_-7H_2_O 0.01 g, agar 18g and distilled water to 1 l (pH 7.2) was used for isolation of actinomycetes [11]. This medium was supplemented with Ampicillin (20 μg/ml) and Fluconazol (25 μg/ml) to inhibit the growth of bacterial and fungal contaminants respectively. The International Streptomyces Project (ISP-2) medium (malt extract 10 g, yeast extract 4 g, glucose 4 g, agar 20 g and distilled water to 1 l (pH 7.3)) was used for purification of actinomycetes isolates [12]. The antimicrobial activity of the isolated actinomycetes was determined in three media: CSA, Bennett's agar medium (yeast extract 1 g, beef extract 1 g, casamino acids 2 g, glucose 10 g, agar 15 g and distilled water to 1 l (pH 7.3) [13] and Mueller-Hinton (MH) agar medium containing beef extract 2 g, acid hydrolysate of casein 17.5 g, soluble starch 1.5 g, agar 17 g and distilled water to 1 l (pH 7.4).

**Strains:** the strains tested (Table 1) were cultivated on Luria Bertani (LB) medium (peptone 10 g, yeast extract 5 g, sodium chloride 10 g and distilled water to 1 l) for bacteria and Sabouraud medium (Casein 5 g, meat extract 5 g, glucose 40 g, agar 15 g and distilled water to 1 l) for yeast at 30°C.

**Table 1:** Strains used in this study

Strains		Source
**Gram-negative bacteria**	*Dickeya solani* IP2222	CNRS- Paris, France
	*Pectobacterium brasiliensis* 13471a	
	*Escherichia coli* K12	Our laboratory’s collection
	*Proteus mirabilis*	
	*Pseudomonas aeruginosa* CECT118	
**Gram-positive bacteria**	*Listeria innocua* CECT4030	
	*Staphylococcus aureus* CECT976	
	*Bacillus subtilis* DSM 347	
**Yeast**	*Candida alibicans*	

**Isolation of actinomycetes:** 10 g of each soil sample were suspended in 100 ml of sterile physiologic water (0.9% NaCl in distilled water). Heat treatment was performed in a water bath at 50°C for 60 min under agitation [14]. Serial 10-fold dilutions of the samples were prepared with sterile 0.9% saline. Dilutions were then plated in triplicate on CSA and incubated for 4 to 6 weeks at 28°C.

**Purification and conservation of actinomycetes isolates:** actinomycetes isolates were recognized by their morphological aspects and by microscopic observation. Isolates were purified by successive streaking on ISP2 medium. They were stored in 20% glycerol at -80°C and/or maintained at 4°C for less than 1 month.

**Antimicrobial activity of actinomycetes isolates:** antimicrobial activities of pure isolates were determined using the double layer agar method test according to the protocol described by Ouhdouch [15]. Pure actinomycetes isolates were spotted on each one of the three tested culture media: CSA, Bennett and Mueller Hinton. After incubation at 30°C or at 37°C for 7 days, the plates were exposed to chloroform vapor for 40 min. Emergent colonies were then covered with a 0.6% agar layer of Bennett's medium, previously seeded with one of the test strains (3.10^+8^ - 4.10^+8^ CFU/ml) and incubated for 48 h at 30°C or at 37°C. Antimicrobial activity was evaluated by measuring the inhibition zone. Each test was carried out in triplicate. For the negative control, the tested strains were grown in the same culture medium without inoculation with actinomycetes.

## Results

**Soil chemical analysis:** pH values obtained for two sites are greater than 7, which qualifies our biotope in the category of calcareous soils (alkaline). Also, the results obtained show that the second site is very salty compared to the first site, which is qualified salty (Table 2).

**Table 2:** Chemical analysis of soil samples

	pH	EC (dS/m) 25°C	Salinity
**Site N°1**	8.80±0.10	1.78±0.10	Salty
**Site N°2**	9.30±0.10	2.45±0.10	Very salty

**Figure 2:**
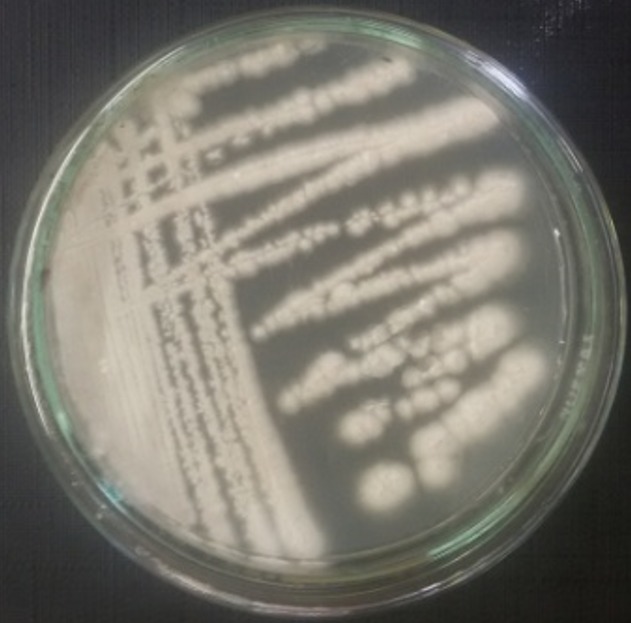
Example of colonie of actinomycete purified on ISP-2 medium

**Isolation of actinomycetes:** a total of 22 isolates (S1-S22) were obtained on CSA medium from the two sites, 13 isolates from the first site and 9 from the second site. In site 1, green isolates are dominant. However, in site 2, white isolates are dominant (Table 3). Figure 2 shows the aspect of actinomycetes isolates developed on ISP-2 medium after 4 weeks at 30°C.

**Table 3:** Number and color of actinomycetes isolates

Color	Number of Actinomycetes isolates	
	Site N°1	Site N°2
White	3	4
Green	8	1
Gray	2	2
Brown	-	1
Pink	-	1
	Total: 22	

**Figure 3:**
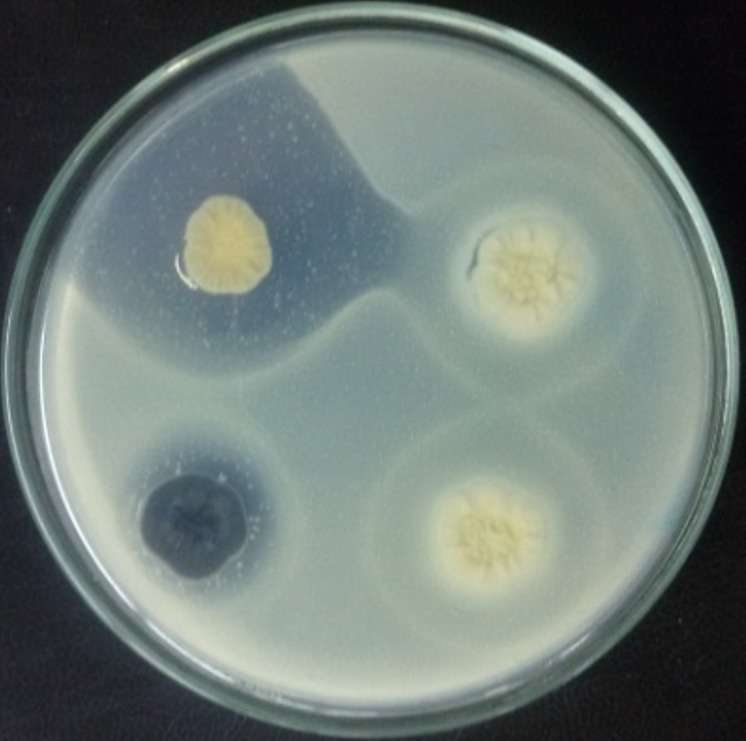
Example of the inhibition zones of actinomycetes against proteus mirabilis in Bennett´s agar medium

**Antimicrobial activity of actinomycetes isolates:** antimicrobial activity was evaluated by measuring the inhibition zone around the actinomycetes colonies. An example of an inhibition zone is shown in Figure 3. Screening results of actinomycetes isolates for the production of antimicrobial substances, on three culture media and at two incubation temperatures, are shown in Table 4 and Table 5.

**Table 4:** Antimicrobial activity of actinomycetes isolates incubated at 30°C for 7 days

Actinomycetes isolates		Inhibition zone (ø, mm)		
Site	code of isolate	Culture media		
		CSA	Bennett	Mueller Hinton
**1**	**S1**	0	20.3±0.5 against S.a	0
	**S2**	0	17.3±0.5 against C.a	0
	**S4**	0	7.3±0.5 against P.m	0
	**S5**	30.3±1.5 against C.a	0	0
	**S6**	0	28±1 against P.m	0
	**S7**	4±1 against L.i	14.3±0.5 against C.a	0
	**S8**	19±1 against C.a	17.3±1.1 against C.a	0
	**S9**	11.6±1.5 against C.a	0	0
	**S10**	0	13±1 against E.c	0
	**S11**	52.3±2.5 against L.i	0	0
	**S12**	23±1 against P.m	4.3±0.5 against D.s	0
	**S13**	13±1 against P.m	31.3±1.5 against P.m	0
**2**	**S15**	0	31.3±1.5 against P.m	0
	**S17**	0	0	3.6±0.5 against P.b
	**S18**	23±1 against P.m	7±1 against L.i	3.3±0.5 against P.b
	**S20**	16±1 against P.m	0	0
	**S21**	0	0	5.3±0.5 against P.b
	**S22**	13.6±1.5 against P.m	0	0

0: not active

Inhibition zone (mm) (ø Inhibition zone − ø colony = ø, mm)

All values are means of three replications with standard deviation

E. c: *Escherichia coli*, P. m: *Proteus mirabilis*, D. s: *Dickeya solani*, P. b: *Pectobacterium brasiliensis*,

S. a : *Staphylococcus aureus*, L. i : *Listeria innocua*, C. a: *Candida albicans*

**Table 5:** Antimicrobial activity of actinomycetes isolates incubated at 37°C for 7 days

Actinomycetes isolates		Inhibition zone (ø, mm)		
Site	code of isolate	Culture media		
		CSA	Bennett	Mueller Hinton
**2**	**S17**	3.3±0.5 against B.s	16.6±1.1 against C.a	15.6±0.5 against P.m
	**S18**	0	0	14.6±0.5 against P.m
	**S20**	0	7.3±0.5 against D.s	0
	**S21**	8±1 against B.s	16.6±0.5 against C.a	0

0 : not active

Inhibition zone (mm) (ø Inhibition zone − ø colony = ø, mm)

All values are means of three replications with standard deviation

B.s: *Bacillus subtilis*, C. a: *Candida albicans*, D. s: *Dickeya solani*, P. m: *Proteus mirabilis*

**Effect of incubation temperature on antimicrobial substances production:** among 22 actinomycetes isolated from both sites and grown at 30°C, 18 isolates seemed to inhibit the growth of at least one microorganism (≈ 82%) (Table 4). On the other hand, when grown at 37°C, most of them aren't even capable to grow on all three-culture media and only 4 isolates (≈ 18%) showed the ability of producing antimicrobial substances (Table 5).

**Effect of culture medium on antimicrobial substances production:** screening results of actinomycetes isolates incubated at 30°C producing antimicrobial substances showed that from the 22 isolates, 11 (50%) are capable of producing antimicrobial substances on Bennett medium, 10 (> 45%) on CSA medium and only 3 on MH medium (> 13%) (Table 4, Table 5).

## Discussion

Actinomycetes are known as versatile producers of antimicrobials active metabolites [16]. Screening has always been the essential method for isolation of new antimicrobial molecules. Several parameters can condition the synthesis and the production of these antimicrobial molecules by the actinomycetes such as the composition of the culture medium, the temperature and the incubation time [17]. Exploitation of particularly extreme sites is very interesting to isolate new species of actinomycetes producing the new antimicrobial molecules. The soil of salterns was chosen as a biotope for the isolation of actinomycetes and for studying their antimicrobial activity in three culture media (Casein Starch Agar, Bennett and Mueller-Hinton) at two incubation temperatures (30°C and 37°C). From the obtained results, it is found that the production of antimicrobial substances by the isolated actinomycetes is influenced by the composition of the culture medium. All the isolates of actinomycetes arrive to grow on the three media, however Bennett´s medium offered the nutritional requirements of these isolates and therefore to show highest number of active isolates. Also, actinomycetes isolates S17 and S21 are active only on Mueller Hinton medium. However, for the same actinomycete isolate, the composition of the culture medium influences the nature of the microorganisms that it inhibits with variable inhibition power: isolate S7 inhibits *Listeria innocua* on CSA medium and inhibits *Candida albicans* on Bennett medium, isolate S12 inhibits *Proteus mirabilis* on CSA medium and inhibits *Dickeya solani* on Bennett medium and isolate S18 inhibits *Proteus mirabilis* on CSA medium, inhibits *Listeria innocua* on Bennett medium and inhibits *Pectobacterium brasiliensis* on Mueller Hinton medium.

Other studies have reported that the Gelatin Broth (GB) medium (glycerol 20 g, soluble starch 20 g, peptone 10 g, meat extract 5 g, CaCO3 3 g, distilled water 1 000 ml, Agar 15 g (pH 7.0)) is favorable to the production of antimicrobial agents in contrary to ISP2 medium [18]. Screening on Bennett medium at 30°C showed that among the 22 isolates of acinomycetes, 6 isolates (> 27%) showed antimicrobial activity against Gram-negative bacteria (*Dickeya solani* IP2222, *Escherichia coli* K12 and *Proteus mirabilis*) and 2 isolates (9%) against Gram-positive bacteria (*Staphylococcus aureus* CECT976 and *Listeria innocua* CECT4030). In relation to incubation temperature, 37°C negatively affects the growth of actinomycetes isolates and consequently diminishing the capacity of production of antimicrobial substances. In 2001, Ouhdouch and collaborators reported that Bennett's medium achieved a maximum number of antifungal activities. Also, production tests of antifungal substances at different temperatures (25°C, 30°C, 37°C and 42°C), show that the temperature of 30°C allows a better production of antifungal substances [15].

## Conclusion

Actinomycetes are the most prolific of all microorganisms as producers of antibiotics. Exploitation of particularly extreme new biotopes is recommended to isolate new species of actinomycetes of biotechnological interest. This is the first study carried out on isolation of actinomycetes from soils of saline salterns conducted in Taza region, Morocco. Control of growing conditions and nutritional requirements of these isolates are very important to find new natural substances with valuable biological activities.

### What is known about this topic

- Actinomycetes are the main source of the vast majority of bioactive secondary metabolites;
- Actinomycetes are produced many antibiotics, that are best recognized and most valuable. These antibiotics include amphotericin, nystatin, chloramphenicol, gentamycin, erythromycin, vancomycin, tetracycline, novobiocin, neomycin, etc;
- The search for new bioactive molecules from actinomycetes remains a priority objective to make the rapid development of microbial resistance to multiple drugs and their undesirable side effects.

### What this study adds

- The isolation of actinomycetes from unexplored ecosystems is one of the most attractive sources for the search for new bioactive metabolites;
- The production of secondary metabolites by actinomycetes depends on the choice of incubation temperature, the use of suitable culture media and the nature of the target pathogen.

## Competing interests

The authors declare no competing interests.
